# Construction of a large collection of small genome variations in French dairy and beef breeds using whole-genome sequences

**DOI:** 10.1186/s12711-016-0268-z

**Published:** 2016-11-15

**Authors:** Mekki Boussaha, Pauline Michot, Rabia Letaief, Chris Hozé, Sébastien Fritz, Cécile Grohs, Diane Esquerré, Amandine Duchesne, Romain Philippe, Véronique Blanquet, Florence Phocas, Sandrine Floriot, Dominique Rocha, Christophe Klopp, Aurélien Capitan, Didier Boichard

**Affiliations:** 1 GABI, INRA, AgroParisTech, Université Paris-Saclay, 78350 Jouy-en-Josas, France; 2 Allice, Maison Nationale des Eleveurs, 75012 Paris, France; 3 GMA, INRA, Université de Limoges, 87060 Limoges Cedex, France; 4 GenPhySE, INRA, INPT, ENVT, Université de Toulouse, Castanet Tolosan, France; 5 SIGENAE, UR 875, INRA, 31362 Castanet-Tolosan, France

## Abstract

**Background:**

In recent years, several bovine genome sequencing projects were carried out with the aim of developing genomic tools to improve dairy and beef production efficiency and sustainability.

**Results:**

In this study, we describe the first French cattle genome variation dataset obtained by sequencing 274 whole genomes representing several major dairy and beef breeds. This dataset contains over 28 million single nucleotide polymorphisms (SNPs) and small insertions and deletions. Comparisons between sequencing results and SNP array genotypes revealed a very high genotype concordance rate, which indicates the good quality of our data.

**Conclusions:**

To our knowledge, this is the first large-scale catalog of small genomic variations in French dairy and beef cattle. This resource will contribute to the study of gene functions and population structure and also help to improve traits through genotype-guided selection.

**Electronic supplementary material:**

The online version of this article (doi:10.1186/s12711-016-0268-z) contains supplementary material, which is available to authorized users.

## Background

In recent years, advances in high-throughput sequencing technologies have offered the opportunity to partially or completely re-sequence genomes, in a relatively cost-effective manner. The availability of whole-genome sequence (WGS) data for an increasing number of individuals offers new opportunities to study genetic variations at the genomic level with unprecedented accuracy.

In the past few years, several whole-genome sequencing studies have been carried out in different dairy and beef cattle breeds and identified a huge number of single nucleotide polymorphisms (SNPs) and small insertions and deletions (InDels) [1–5]. To date, the Ensembl (http://www.ensembl.org) short variation database contains over 99 million SNPs and InDels identified in several cattle breeds. During the first phase of the 1000 bull genomes project, the genomes of 234 bulls were sequenced, which has enabled the identification of over 28 million reliable SNPs and InDels [5]. Only 13 French bulls were included in this phase.

In this work, we performed a large-scale study to investigate both SNPs and small InDels in whole-genome sequencing data for 274 animals from several major French dairy and beef breeds. The collection of genome variations reported in this study will be useful to study their potential links with the genetic variability of economically important traits.

## Methods

### Animal ethics

No animal experimentation was used in this study, since no new tissue samples were collected. All whole-genome sequence data used in this study were already available in our laboratory and were produced as previously described [1].

### Whole-genome sequencing and sequence alignment to the reference

The whole genome of 274 animals corresponding to both French dairy and beef breeds (Table 1) were used for 2 × 100 bp paired-end sequencing on an Illumina HiSeq 2000 with a TruSeq SBS v3-HS Kit (Illumina).

**Table 1:** Number of animals used per breed

Breed	Number of animals
Abondance	1
Aubrac	8
Brown Swiss	3
Salers	3
Tarentaise	1
Limousine	20
Simmental	1
Charolaise	34
Rouge des Prés	5
Montbéliarde	59
Normande	43
Vosgienne	4
Holstein	63
Parthenaise	2
Blonde d’Aquitaine	26
Cross-breed	1

This table lists the distribution in each breed of the 274 sequenced animals

Sequence alignments were carried out using the Burrows-Wheeler Alignment tool (BWA-v0.6.1-r104) [6] with the aln option with default parameters for mapping reads to the UMD3.1 bovine reference genome [7]. Potential PCR duplicates, which can adversely affect the variant calls, were removed using the MarkDuplicates tools from the Picard package version 1.4.0 [8]. Only properly paired reads with a mapping quality of at least 30 (−q = 30) were retained. The resulting BAM files were then used for all subsequent analyses.

### Identification of small insertions and deletions

Small genomic variations were detected using the Genome Analysis Tool Kit 2.4–9 (GATK) version and GATK-UnifiedGenotyper as SNP caller [9]. Prior to variant discovery, reads were subjected to local realignment, coordinate sorting, quality recalibration, and removal of PCR duplicates. In the GATK analysis, we used a minimum confidence score threshold of Q30 with default parameters. We also used multi-sample variant calling in order to distinguish between a homozygous reference genotype and a missing genotype in the analyzed samples.

### Variant annotation

All variants were annotated with the Ensembl variant effect predictor (VEP) pipeline v81 [10] based on the Ensembl version 81 transcript set and using dbSNP build 143. The effect of the amino acid changes was predicted using SIFT [11, 12], a sequence homology-based tool that can determine whether an amino acid substitution in a protein is deleterious or tolerant.

### Functional characterization of protein-coding genes with LoF variants

A set of 8337 gene products was used for gene ontology (GO) enrichment and functional analyses, using the GO [13] and the KEGG (Kyoto Encyclopedia of Genes and Genomes) [14] database resources. The Cytoscape [15] ClueGO plugin [16] was used to identify the biological functions to which genes contribute. The enrichment of biological terms and groups were set as follows. First, we used the enrichment tests based on the hyper-geometric distribution. Second, we set the statistical significance to 0.05 (p  ≤  0.05), and we used the Benjamini-Hochberg adjustment to correct the p value for the terms and the groups created by ClueGO. Third, we used fusion criteria to reduce the redundancy of related terms that have similar associated genes. Finally, we set the Kappa-statistics score threshold to 0.6.

Gene Ontology (GO) enrichment was also performed using the MouseMine analysis tools available at the MGI international database resources (http://www.mousemine.org/mousemine/begin.do).

### Validation of LoF variants by high-throughput genotyping

The efficiency of our calling approach and the relevance of the resulting variants were assessed by genotyping a selected panel containing 304 heterozygous deleterious missense and loss-of-function SNPs for which no homozygous individual for the alternative allele was observed in our population. Genotyping was performed using the already available Illumina BovineLD custom BeadChip [17] and a panel of 172,416 beef and dairy cattle animals (Table 2).

**Table 2:** Total number of animals genotyped using the Illumina Bovine low density BeadChip

Breed	Number of animals
Abondance	39
Brown Swiss	627
Tarentaise	49
Limousine	2084
Simmental	2
Montbéliarde	55,382
Normande	20,697
Holstein	90,970
Blonde d’Aquitaine	2566

This table summarizes the number and the distribution in each breed of the animals genotyped using the Illumina bovine low density BeadChip

## Results and discussion

### Whole-genome sequencing and read mapping

Two hundred and seventy four animals corresponding to both French dairy and beef breeds were selected for whole-genome sequencing (Table 1), of which 62 whole-genome sequences were already published [1]; the Illumina short reads are available at the European Nucleotide Archive (ENA) with study accession number PRJEB9343 (http://www.ebi.ac.uk/ena/data/view/PRJEB9343). Overall, 103 billion raw paired-end reads 100-bases long were generated, which resulted in over ten thousand gigabases of data. On average, 95% (from 56 to 99%) of the paired-end reads were properly aligned on the UMD3.1 bovine reference genome (see Additional file 1), which is in agreement with previous studies [1, 18]. The average genome-wide sequence coverage from the mapped reads was 13.8× and ranged from almost 5× to around 36× across the different genomes, with 236 samples sequenced at least at 10-fold average coverage (see Additional file 1).

### Identification of SNPs and small InDels

A search for small genome variations with the GATK-UnifiedGenotyper software resulted in the identification of 28,164,518 variants, of which 25,210,883 were SNPs, 1769,413 small deletions and 1184,222 small insertions. Almost 87% of the deletions and 93% of the insertions identified in our study were 1 to 3 bp long (see Additional file 2). The largest deletions and insertions identified were respectively 58 and 29 bp long (see Additional file 2). Overall, 73% of the identified variants (20,647,361) were known in the Ensembl variation 83 database (build 143). The remaining 27% were considered as novel variants and should contribute to better highlight the genetic variability in cattle.

A total of 146,944 genome variants were identified as bi-allelic in our dataset but contained more than two alleles in the Ensembl variation 83 database. Of these 146,944 genome variants, only 95 positions that displayed a single variant type in our dataset overlapped with multiple variant types in the Ensembl variation 83 database. For the remaining 146,849 positions, a single variant type was observed in both databases, of which 129,356 (88.1%) SNPs and 17,493 (11.9%) InDels were identified in our dataset. Among the 129,356 discrepant SNPs, 99.3% (128,407) were reported to be tri-allelic SNPs and only 0.7% (949) corresponded to InDels in the Ensembl variation 83 database. Of the 17,493 discrepant InDels, 67.3% (11,770) corresponded to tri-allelic SNPs and 32.7% (5723) were also InDels but with multiple alleles in the Ensembl variation 83 database. In addition, we identified 88,289 positions that displayed one type of variant (i.e. SNP or InDel but not both) in our dataset but which overlapped with multiple variant types in the Ensembl variation 83 database. We also identified 517,417 variants for which the alleles differed between our dataset and the Ensembl 83 variation database. These inconsistencies could be partly explained by the use of different variant calling algorithms. Indeed, a previous study in Danish Holstein dairy cattle also reported similar inconsistencies [3]. In that study, genotype accuracy was assessed for 15 variants for which samtools-derived genotypes differed from those predicted by GATK. Their results revealed that GATK provided more accurate genotype calls than samtools.

### Evaluation of sequencing genotypes

**Fig. 1:**
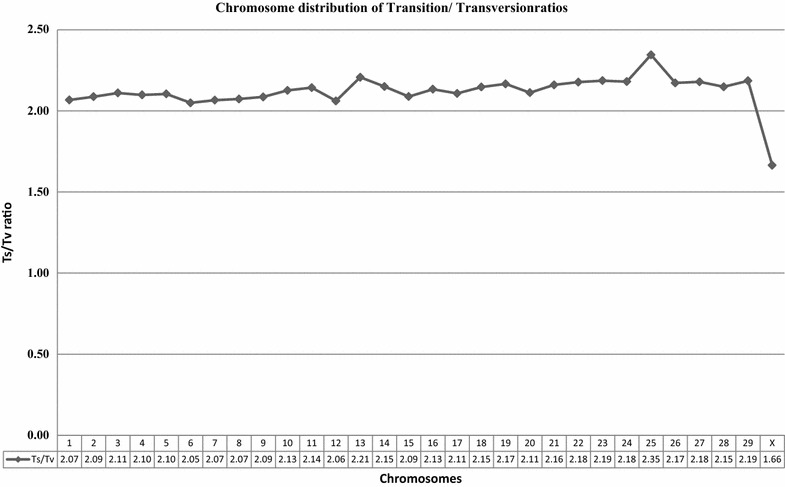
Chromosome distribution of transition/transversion ratios. Average Ts/Tv ratios over all animals were plotted for all autosomes and the X chromosome

To evaluate the quality of our sequencing data-derived genotypes, we performed three different analyses. First, we used the ratio of transitions over transversions (Ts/Tv) as a diagnostic measure to assess the quality of our sequencing data. The average Ts/Tv ratio observed in our whole-genome sequencing data was 2.12 and ranged from 2.05 on BTA6 to 2.35 on BTA25 (Fig. 1). This average rate is within the same range as those observed in other species. For example, in human whole-genome sequence data, the genome-wide Ts/Tv ratio ranged from 2.0 to 2.2 [19, 20]. In mouse and pig, similar ratios were reported i.e. about 2.0 [21] and 2.04 ± 0.28 [22], respectively. DePristo et al. [19] indicated that the Ts/Tv ratio should be around 2.1 for whole-genome sequencing and that lower ratios may indicate that the sequencing data includes false positives caused by random sequencing errors. Therefore, the Ts/Tv ratio estimated in our study is indicative of good sequencing data quality.

**Fig. 2:**
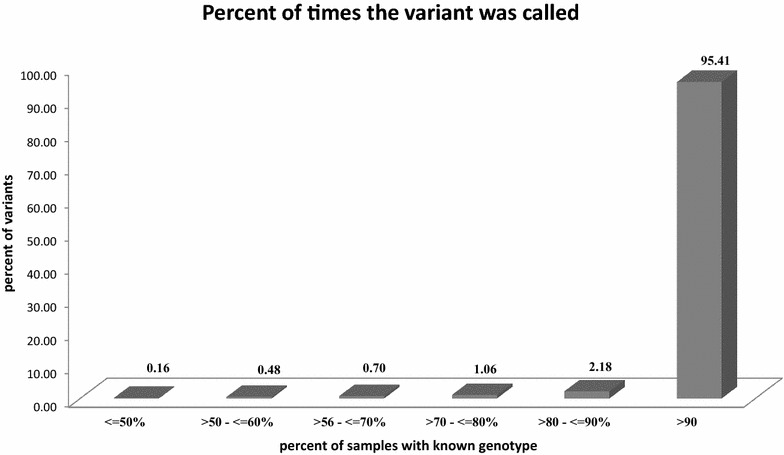
Percent of times the variant was called. This plot summarizes the number of times the variant was called over the number of samples with known genotypes

Second, we measured the call rate by estimating the percentage of samples presenting a known genotype for each variant. On average, 95% of the variants were called in more than 90% of the samples with 13% (3,655,506) of the variants being genotyped in all 274 samples (Fig. 2).

**Fig. 3:**
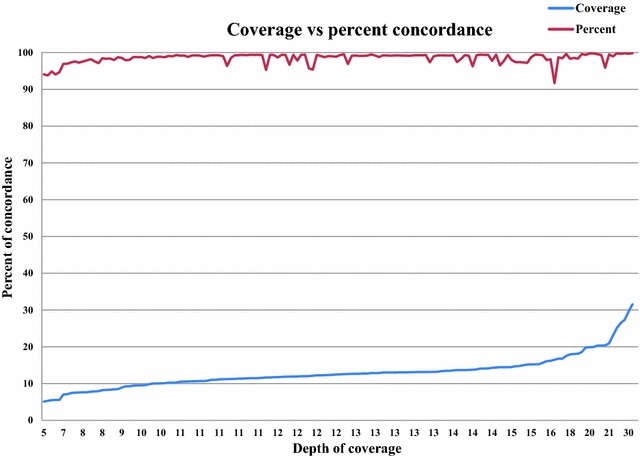
Coverage versus percent concordance. This plot summarizes the sequence coverage versus the accuracy percent between sequences derived-genotypes and SNP-array ones

Third, we compared our sequencing data-derived genotypes to SNP array-derived genotypes using the Illumina High-Density (HD) Bovine SNP BeadChip^®^ which includes 777,962 SNPs [23]. Overall, both genotyping data sources were available for 152 samples. The average genotype concordance rate was around 99.1% and ranged from 91.7 to 99.8% (see Additional file 3). We also observed a dependency of chip genotype concordance on sequencing depth (see Additional file 3; Fig. 3). Lower accuracy rates were found for samples with a low depth of coverage (less than 10×). For 21 samples, the concordance rate was less than 98% but their depth of coverage was higher than 11×. Of these 21 samples, 20 had a concordance rate between 95 and 97% and were considered as acceptable. The observed lower concordance rates could be partly due to lower sequence data quality compared to the rest of our sample set.

The low missing rate and high concordance rate observed in our study can be explained by the good average genome-wide sequence coverage of the mapped reads in our data. Indeed, more than 86% of our samples were sequenced at least at an average 10-fold coverage. Another reason is the efficiency of our variation calling strategy.

### Functional annotation of small genome variants

Functional annotation of the identified small genome variants was carried out using the Ensembl VEP annotation software [10]. Overall, 66% of the annotated variants were located in intergenic regions and almost 30% were identified within gene intronic sequences (Table 3). The remaining 4% were located within gene-coding, upstream and downstream regions. Of these, 85,038 variants were located within the 5′ or 3′ untranslated regions (UTR), 171 were located within genes coding for micro RNAs (miRNAs), 96,711 missense mutations were identified within gene coding regions, 358 InDels were predicted to cause inframe insertions and 814 InDels were predicted to cause inframe deletions.

**Table 3:** Results of functional annotation by VEP

Functional class	All	SNP	InDels
3′ UTR variant	70,139	61,080	9059
5′ UTR variant	14,899	13,696	1203
Frameshift variant	2287	0	2287
Inframe deletion	814	0	814
Inframe insertion	358	0	358
Splice acceptor variant	649	510	139
Splice donor variant	1471	1378	93
Start lost	169	169	0
Stop gained	1159	1139	20
Stop lost	68	67	1
Mature miRNA variant	171	135	36
Intron variant	8,446,403	7,513,594	932,809
Downstream gene variant	1,335,987	1,179,502	156,485
Intergenic variant	18,568,837	16,664,272	1,904,565

This table summarizes the functional classification of all variants reported in this study

Overall, we identified 2120 variants that affected splice sites. These included 1471 splice donor and 649 splice acceptor site variants. In addition, 1159 variants were predicted to create a premature stop codon and 68 to disrupt a termination codon. Around 2287 InDels were predicted to cause a frameshift in coding sequences which were considered as loss-of-function (LOF) variants and may result in reduced or complete inactivation of protein functions by disrupting either the protein-coding gene itself or genetic regulatory elements. These LOF variant candidates are of particular interest since they might have effects on economically important traits.

Among the annotated deleterious missense and LOF variants, we identified several mutations that were previously reported to be associated with dairy and beef traits in cattle. For example, the amino acid change of phenylalanine to leucine at position 94 (F94L) of the myostatin (MSTN) protein was identified in 31 samples, among which six animals were heterozygous (three Charolaise, two Aubrac and one Rouge-des-Prés) and 25 were homozygous (19 Limousine and six Aubrac) for this locus. We also observed the *MSTN* pQ204* mutation in eight samples, all of which corresponded to the Charolaise breed and all animals were heterozygous. Both F94L and Q204* substitutions are associated with double muscling phenotypes in Limousine [24] and Charolaise [25] cattle, respectively.

The F279Y mutation within the *growth hormone receptor* (*GHR*) gene was observed in 35 samples corresponding to 29 dairy and six beef cattle animals (four Blonde d’Aquitaine, one Brown Swiss, one Charolaise, two Montbéliarde, five Normande and 22 Holstein) with the highest frequency observed in the Holstein breed (19 heterozygous and three homozygous individuals for the alternative allele). This SNP is located on BTA20 and has been shown to be associated with milk yield and composition [26, 27], feed intake, feed conversion efficiency and body energy traits [28].

### Missense and LOF variants for which no homozygous individuals for the alternative allele are observed

Further analysis of the annotated variants revealed the presence of 14,469 missense and LOF variants with a significant biological impact based on SIFT predictions and for which no homozygous animal carrying the alternative allele was observed among the 274 WGS (see Additional file 4). These were subsequently considered as our study panel in the rest of this paper.

This study panel contains 772 frameshift variants, 12,008 missense mutations with a deleterious effect predicted by SIFT with a score between 0 and 0.05, 67 start-lost variants, 583 stop variants (25 stop-lost and 558 stop-gained) and 1039 splice variants (264 splice-acceptor and 775 splice-donor variants).

The genotype distribution of our study panel revealed that seven frameshift variants were breed-specific (Table 4). Integrated Genome Viewer (IGV) visualization and inspections of BAM files for animals carrying these mutations revealed that four of the seven frameshift mutations were spurious variant calls (results not shown). The three remaining frameshift variants could be visualized and confirmed by IGV and were therefore considered as true variants. First, a five nucleotide insertion (-/CACGT) at position 66,552,044 on BTA1 was identified in two Blonde d’Aquitaine animals. This frameshift mutation was absent in both the Ensembl database and in the most recent 1000 bull genomes project dataset which contains small genomic variations for 1577 animals corresponding to 48 different breeds (Daetwyler HD, personal communication). This mutation affects the sequences of *A*-*family polymerase theta* (*POLQ*) gene by producing a frameshift insertion between amino acids number 2728 and 2729 just before the termination site. It induces a frameshift in the open reading frame which results in the addition of ten new amino acids followed by a new downstream termination site. The *POLQ* gene has been identified in several other species and was reported to play a major role in the DNA repair mechanism of double strand breaks (DSB) by alternative end-joining (alt-EJ; also called alternative non homologous end-joining (alt-NHEJ) or microhomology-mediated end joining)) [29–33]. Unlike the classical non homologous end-joining (c-NHEJ) mechanism, alt-EJ depends on resection of DNA ends to find microhomologies, which results in larger deletions and insertions [34, 35]. Inhibition of POLQ functions in mice were reported to be associated with chromosome instability phenotypes [36]. In mammalian cells, POLQ promotes the formation of chromosomal translocations and is essential for survival when the homology-directed repair (HDR) mechanism is impaired [31], which suggests that this mutation may cause embryonic lethality in cattle.

**Table 4:** Distribution of LoF and deleterious variants

	Stop lost	Splice acceptor	Start lost	Frameshift	Missense deleterious	Stop gained	Splice donor
Holstein	1	15	10	0	1	5	18
Abondance	1	0	1	0	43	1	0
Cross-breed	0	0	0	1	10	1	0
Brown Swiss	0	6	1	0	4	3	3
Salers	0	1	0	0	47	1	2
Montbéliarde	1	1	3	0	705	34	18
Vosgienne	0	4	0	0	4	9	3
Normande	0	11	3	0	626	1	1
Simmental	0	2	0	0	33	3	1
Limousine	0	11	1	0	18	1	14
Charolaise	1	15	6	5	17	35	21
Parthenaise	0	0	0	0	1	9	2
Rouge des Prés	1	1	0	0	37	3	1
Tarentaise	0	1	0	0	54	0	0
Blonde d’Aquitaine	2	13	3	1	572	31	16
Aubrac	0	8	1	0	193	10	2

This table summarizes the distribution of LoF and deleterious variants in each breed and for each functional annotation class

The two other frameshift mutations were identified in the Charolaise breed. The first one is a GACC insertion at position 149,472 on BTA19 and is located within an olfactory receptor gene coding sequence (*ENSBTAG00000045560*). This variant was identified in three samples in our dataset and is also present in the Ensembl database. It leads to a frameshift mutation within the 7tm_1 (PF000001) pfam domain at amino acids 81 and 82 and creates 39 new amino acids followed by a termination site, thus producing a truncated protein, which contains only 26% (82 of 311 amino acids) of the wild type protein. The second frameshift mutation is a four nucleotide (-/AGTT) insertion identified at position 21,913,213 on BTA18. It was identified in two samples in our panel but it is absent in the Ensembl database. It is located within the *retinoblastoma*-*like 2* (*RBL2*) coding gene region and leads to a frameshift mutation within the RB_B box (PF01857 pfam domain) at amino acids 890–891, thus introducing 26 new amino acids before creating a premature termination site. Thus, a truncated protein representing only 78% (890/1140 amino acids) of the wild type protein is produced. RBL2, also called pRb2/p130, is a member of the retinoblastoma family of tumor suppressors [37] and its expression was reported to be altered in several cancer types [38–40]. RBL2 interacts with the E2F4 and E2F5 transcription factors and results in negative regulation of the cell cycle [41]. It is also involved in many other cellular processes, such as regulation of apoptosis and differentiation [37] and control of the length of telomeres [42].

Finally, we identified the p.Q579* mutation within the *APAF1* gene (HH1: Holstein Haplotype 1), the p.N290T deleterious missense mutation within the *GART* gene (HH4), the p.V180F deleterious missense mutation within the *SLC35A3* gene (CVM: complex vertebral malformation), the p.Q52* stop-gained variant within the *SHBG* gene (MH1: Montbéliarde Haplotype1) and the R12* stop-gained variant within the *SLC37A2* gene (MH2). All these substitutions are specific to the Holstein (HH1, HH4 and CVM) and Montbéliarde (MH1 and MH2) breeds, respectively and are considered to be strong candidate mutations for embryonic lethal defects [43].

### Gene ontology and pathway analysis

In order to gain additional insight into the biological pathways and molecular functions that are affected by these variants, we performed a gene ontology (GO) enrichment and functional analysis using 8337 known Ensembl ID-associated genes retrieved from our variant annotation study (see Additional file 5). Several GO terms were significantly over-represented. For example, the six most enriched GO categories corresponding to biological processes were related to the regulation of GTPase-, Ras-, and Rho-mediated signal transduction. The three most enriched GO categories corresponding to cellular components were related to cytoskeleton and myosin complex and the five most enriched GO categories corresponding to molecular functions were related to ATP binding, adenine nucleotide binding, ATPase activity, motor activity and ribonucleotide binding.

### Experimental validation of the study panel by high-throughput genotyping

Previous studies reported a significant rate of false positive calls among deleterious missense and loss-of-function variants [3, 44]. Thus, the efficiency of our calling approach and the relevance of the resulting variants were assessed by genotyping a selected panel containing 304 heterozygous deleterious missense and loss-of-function mutations for which no homozygous individual for the alternative allele was observed in our population. They were also selected based on their mapping quality (above 50) and their calling quality (above 30) scores. Genotyping was performed using the already available Illumina BovineLD custom BeadChip [17] and a panel of 172,416 animals corresponding to both beef and dairy cattle breeds (Table 2).

Overall, 276 (~91%) SNPs were polymorphic in all genotyped animals and were considered as true variants (see Additional file 6). Among these, 61 SNPs were private and were polymorphic only in one breed. Thus, they were considered as breed-specific variants i.e. two in Brown Swiss, three in Limousine, 12 in Montbéliarde, 27 in Normande, 16 in Holstein and one in Blonde d’Aquitaine. For 51 polymorphic SNPs, we observed only two genotypes. No homozygous individual for the alternative allele was observed among all genotyped samples. For these 51 variants, we determined the expected proportions of homozygous individuals for the alternative allele in each breed and then calculated the significance probability (p value) from the binomial distribution, with event probability equal to zero (which corresponded to the proportion of observed homozygous individuals for the alternative allele), and the number of observations was equal to the  number of animals in each breed. For 41 of the 51 variants, there was no significant difference between the expected and the observed proportions (see Additional file 6). However, for the other 10 variants, the expected proportion was significantly different from the observed proportion in at least one breed (see Additional file 6). These corresponded to nine missense deleterious mutations and one LOF variant. This latter one corresponded to the p.Q579* mutation within the *APAF1* gene (HH1: Holstein Haplotype 1) which was previously reported as a strong candidate mutation for embryonic lethal defects [43]. As expected, significant differences between the observed and estimated proportions of homozygous individuals for the alternative allele were only observed in the Holstein breed. Two other deleterious missense mutations were also located within *CBX3* (*chromobox protein homolog 3*) and *RBBP5* (*RB binding protein 5, histone lysine methyltransferase complex subunit*) genes which are known to be associated with male germ cell survival and spermatogenesis [45] and sterility [46], respectively.

The 51 SNPs for which only two genotypes were observed were located within 42 known gene coding regions. Thus, these genes were used to carry out gene ontology (GO) and mammalian phenotype ontology (MPO) enrichment analyses using the MouseMine analysis tools (see Additional file 7). The most significant enriched MPO categories were related to abnormal nervous system morphology and phenotype, preweaning lethality, and abnormal brain development (see Additional file 7, sheet1). However, no significant GO category enrichment was obtained (see Additional file 7, sheet 2). It will be very interesting to study the effect of these variants on phenotypes of interest in cattle.

## Conclusions

In this study, we performed a pan-genome assessment of small genome variations in cattle using whole-genome sequence data. Analysis of WGS data of 274 animals from both dairy and beef cattle breeds allowed the identification of over 28 millions small variations, among which we identified more than 25 million SNPs and around 3 million small insertions and deletions. To assess the quality of both our sequencing data and calling approach, we analyzed the transition to transversion ratio and the call rate, and we also compared the sequence-derived genotypes with array-derived ones. Results from all these analyses confirmed the efficiency of our sequencing data as well as the good quality of our variant calling procedure. Annotation of these variants revealed several deleterious missense and loss-of-function variants, among which we identified several mutations that were previously reported to be associated with either dairy or beef traits. Genotypic and allelic frequency distributions revealed the presence of more than 14,000 heterozygous candidate deleterious and LOF variants that segregated in the absence of individuals homozygous for the alternative allele in our population. Of these, we genotyped 172,416 animals from dairy and beef breeds with a panel of 304 SNPs, using the already available Illumina BovineLD custom BeadChip. Two hundred and seventy-six of these variants (~91%) were polymorphic in at least one breed and, thus, were considered as true variants. For 51 of the 276 polymorphic variants, we did not observe any homozygous individual for the alternative allele. These 51 variants will be useful to study their link with genetic variability of economically-important traits in cattle.

## Additional files

**Additional file 1.** Summary of sequence coverage and sequence alignment to the reference genome. Total and mapped reads, as well as total and percent of correctly mapped reads are indicated for each sample. The estimated sequence coverage was also obtained for each sample.

**Additional file 2.** Size distribution of small insertions and deletions identified by GATK. Total number of small insertions and deletions and their distribution by size are indicated.

**Additional file 3.** Results of genotype accuracy analyses. Genotype accuracy analyses were performed by comparing sequence-derived genotypes and those obtained using the high-density SNP bead chip for 152 samples (39 Holstein, 49 Montbéliarde, 41 Normande and 23 Blonde d’Aquitaine).

**Additional file 4.** List of LoF and deleterious missense variants for which no homozygous individual for the alternative allele was observed in our samples. For each variant, overall and per breed genotype (0/0, 0/1 and 1/1) distribution as well as the frequency for the alternative allele are indicated. The functional annotation results and SIFT predictions are also indicated.

**Additional file 5.** GO enrichment analysis using the Cytoscape ClueGO plugin. The results of GO enrichment analyses were obtained using the overall list of LoF and deleterious missense variants for which no homozygous individual for the alternative allele was observed in our samples. The results for biological processes (BP), cellular components (CP) and molecular functions (MF) are in sheets 1, 2 and 3, respectively.

**Additional file 6.** Frequency of the alternative allele of SNPs in our validation panel. (Sheet 1) Frequency of the alternative allele was calculated using the genotyping data obtained by genotyping all variants of our validation panel with the custom Illumina Bovine low-density BeadChip. Monomorphic variants (homozygous for the reference allele) in each breed are indicated by “0“. Missing genotyping data are indicated by “ng”. The numbers of observed types of genotype are in column 3 and coded as follows: (1) monomorphic variants; (2) and (3) correspond to the 276 polymorphic variants with (2) corresponding to the 51 variants for which no homozygous individual for the alternative allele was observed and (3) corresponding to variants for which we observed the three genotypes. (Sheet 2) Results of the exact binomial distribution test with event probability being set to zero (which corresponds to the number of observed homozygous individuals for the alternative allele), and n  was equal to the  number of animals in each breed (indicated between parenthesis). For the Simmental breed, we had only two genotyped animals and therefore we did not do the binomial distribution test.

**Additional file 7.** Results of GO enrichment. GO enrichment analyses were performed using the MouseMine analysis tools and the results are summarized in sheets 1 and 2. Mammalian phenotype enrichment results are in sheet 1 and GO enrichment results in sheet 2.

## References

[1] Boussaha M, Esquerré D, Barbieri J, Djari A, Pinton A, Letaief R (2015). Genome-wide study of structural variants in bovine Holstein, Montbéliarde and Normande dairy breeds. PLoS One.

[2] Stothard P, Liao X, Arantes AS, De Pauw M, Coros C, Plastow GS (2015). A large and diverse collection of bovine genome sequences from the Canadian cattle genome project. Gigascience.

[3] Das A, Panitz F, Gregersen VR, Bendixen C, Holm LE (2015). Deep sequencing of Danish Holstein dairy cattle for variant detection and insight into potential loss-of-function variants in protein coding genes. BMC Genomics.

[4] Baes CF, Dolezal MA, Koltes JE, Bapst B, Fritz-Waters E, Jansen S (2014). Evaluation of variant identification methods for whole genome sequencing data in dairy cattle. BMC Genomics.

[5] Daetwyler HD, Capitan A, Pausch H, Stothard P, van Binsbergen R, Brøndum RF (2014). Whole-genome sequencing of 234 bulls facilitates mapping of monogenic and complex traits in cattle. Nat Genet.

[6] Li H, Durbin R (2009). Fast and accurate short read alignment with Burrows–Wheeler transform. Bioinformatics.

[7] Zimin AV, Delcher AL, Florea L, Kelley DR, Schatz MC, Puiu D (2009). A whole-genome assembly of the domestic cow, *Bos taurus*. Genome Biol.

[8] Picard Tools—by Broad Institute. http://broadinstitute.github.io/picard/.

[9] McKenna A, Hanna M, Banks E, Sivachenko A, Cibulskis K, Kernytsky A (2010). The genome analysis toolkit: a MapReduce framework for analyzing next-generation DNA sequencing data. Genome Res.

[10] McLaren W, Pritchard B, Rios D, Chen Y, Flicek P, Cunningham F (2010). Deriving the consequences of genomic variants with the Ensembl API and SNP Effect Predictor. Bioinformatics.

[11] Ng PC, Henikoff S (2001). Predicting deleterious amino acid substitutions. Genome Res.

[12] Kumar P, Henikoff S, Ng PC (2009). Predicting the effects of coding non-synonymous variants on protein function using the SIFT algorithm. Nat Protoc.

[13] Ashburner M, Ball CA, Blake JA, Botstein D, Butler H, Cherry JM (2000). Gene ontology: tool for the unification of biology. The Gene Ontology Consortium. Nat Genet.

[14] Kanehisa M, Goto S, Kawashima S, Nakaya A (2002). The KEGG databases at GenomeNet. Nucleic Acids Res.

[15] Shannon P, Markiel A, Ozier O, Baliga NS, Wang JT, Ramage D (2003). Cytoscape: a software environment for integrated models of biomolecular interaction networks. Genome Res.

[16] Bindea G, Mlecnik B, Hackl H, Charoentong P, Tosolini M, Kirilovsky A (2009). ClueGO: a Cytoscape plug-into decipher functionally grouped gene ontology and pathway annotation networks. Bioinformatics.

[17] Boichard D, Chung H, Dassonneville R, David X, Eggen A, Fritz S (2012). Design of a bovine low-density SNP array optimized for imputation. PLoS One.

[18] Kawahara-Miki R, Tsuda K, Shiwa Y, Arai-Kichise Y, Matsumoto T, Kanesaki Y (2011). Whole-genome resequencing shows numerous genes with nonsynonymous SNPs in the Japanese native cattle Kuchinoshima-Ushi. BMC Genomics.

[19] DePristo MA, Banks E, Poplin R, Garimella KV, Maguire JR, Hartl C (2011). A framework for variation discovery and genotyping using next-generation DNA sequencing data. Nat Genet.

[20] Ebersberger I, Metzler D, Schwarz C, Pääbo S (2002). Genomewide comparison of DNA sequences between humans and chimpanzees. Am J Hum Genet.

[21] Lindblad-Toh K, Winchester E, Daly MJ, Wang DG, Hirschhorn JN, Laviolette JP (2000). Large-scale discovery and genotyping of single-nucleotide polymorphisms in the mouse. Nat Genet.

[22] Bianco E, Nevado B, Ramos-Onsins SE, Pérez-Enciso M (2015). A deep catalog of autosomal single nucleotide variation in the pig. PLoS One.

[23] Matukumalli LK, Lawley CT, Schnabel RD, Taylor JF, Allan MF, Heaton MP (2009). Development and characterization of a high density SNP genotyping assay for cattle. PLoS One.

[24] Sellick GS, Pitchford WS, Morris CA, Cullen NG, Crawford AM, Raadsma HW (2007). Effect of myostatin F94L on carcass yield in cattle. Anim Genet.

[25] Grobet L, Poncelet D, Royo LJ, Brouwers B, Pirottin D, Michaux C (1998). Molecular definition of an allelic series of mutations disrupting the myostatin function and causing double-muscling in cattle. Mamm Genome.

[26] Blott S, Kim JJ, Moisio S, Schmidt-Küntzel A, Cornet A, Berzi P (2003). Molecular dissection of a quantitative trait locus: a phenylalanine-to-tyrosine substitution in the transmembrane domain of the bovine growth hormone receptor is associated with a major effect on milk yield and composition. Genetics.

[27] Viitala S, Szyda J, Blott S, Schulman N, Lidauer M, Mäki-Tanila A (2006). The role of the bovine growth hormone receptor and prolactin receptor genes in milk, fat and protein production in Finnish Ayrshire dairy cattle. Genetics.

[28] Banos G, Woolliams JA, Woodward BW, Forbes AB, Coffey MP (2008). Impact of single nucleotide polymorphisms in leptin, leptin receptor, growth hormone receptor, and diacylglycerol acyltransferase (DGAT1) gene loci on milk production, feed, and body energy traits of UK dairy cows. J Dairy Sci.

[29] Ceccaldi R, Liu JC, Amunugama R, Hajdu I, Primack B, Petalcorin MIR (2015). Homologous-recombination-deficient tumours are dependent on Polθ-mediated repair. Nature.

[30] Koole W, van Schendel R, Karambelas AE, van Heteren JT, Okihara KL, Tijsterman M (2014). A polymerase theta-dependent repair pathway suppresses extensive genomic instability at endogenous G4 DNA sites. Nat Commun..

[31] Mateos-Gomez PA, Gong F, Nair N, Miller KM, Lazzerini-Denchi E, Sfeir A (2015). Mammalian polymerase θ promotes alternative NHEJ and suppresses recombination. Nature.

[32] Roerink SF, van Schendel R, Tijsterman M (2014). Polymerase theta-mediated end joining of replication-associated DNA breaks in *C. elegans*. Genome Res.

[33] Yousefzadeh MJ, Wyatt DW, Takata KI, Mu Y, Hensley SC, Tomida J (2014). Mechanism of suppression of chromosomal instability by DNA polymerase POLQ. PLoS Genet.

[34] Yu AM, McVey M (2010). Synthesis-dependent microhomology-mediated end joining accounts for multiple types of repair junctions. Nucleic Acids Res.

[35] McVey M, Lee SE (2008). MMEJ repair of double-strand breaks (director’s cut): deleted sequences and alternative endings. Trends Genet.

[36] Fernandez-Vidal A, Guitton-Sert L, Cadoret JC, Drac M, Schwob E, Baldacci G (2014). A role for DNA polymerase θ in the timing of DNA replication. Nat Commun.

[37] Indovina P, Marcelli E, Casini N, Rizzo V, Giordano A (2013). Emerging roles of RB family: new defense mechanisms against tumor progression. J Cell Physiol.

[38] Milde-Langosch K, Goemann C, Methner C, Rieck G, Bamberger AM, Löning T (2001). Expression of Rb2/p130 in breast and endometrial cancer: correlations with hormone receptor status. Br J Cancer.

[39] Li Q, Sakurai Y, Ryu T, Azuma K, Yoshimura K, Yamanouchi Y (2004). Expression of Rb2/p130 protein correlates with the degree of malignancy in gliomas. Brain Tumor Pathol..

[40] D’Andrilli G, Masciullo V, Bagella L, Tonini T, Minimo C, Zannoni GF (2004). Frequent loss of pRb2/p130 in human ovarian carcinoma. Clin Cancer Res.

[41] Dyson N (1998). The regulation of E2F by pRB-family proteins. Genes Dev.

[42] Kong LJ, Meloni AR, Nevins JR (2006). The Rb-related p130 protein controls telomere lengthening through an interaction with a Rad50-interacting protein, RINT-1. Mol Cell.

[43] Fritz S, Capitan A, Djari A, Rodriguez SC, Barbat A, Baur A (2013). Detection of haplotypes associated with prenatal death in dairy cattle and identification of deleterious mutations in GART, SHBG and SLC37A2. PLoS One.

[44] Kõks S, Reimann E, Lilleoja R, Lättekivi F, Salumets A, Reemann P (2014). Sequencing and annotated analysis of full genome of Holstein breed bull. Mamm Genome.

[45] Brown JP, Bullwinkel J, Baron-Lühr B, Billur M, Schneider P, Winking H, Singh PB (2010). HP1gamma function is required for male germ cell survival and spermatogenesis. Epigenet Chromatin.

[46] Li T, Kelly WG (2011). Li T, Kelly WG. A role for Set1/MLL-related components in epigenetic regulation of the Caenorhabditis elegans germ line. PLoS Genet.

